# Mogamulizumab and Concomitant Hypofractionated Low-Dose Total Skin Electron Beam Therapy (2 × 4 Gy) in Cutaneous T-Cell Lymphoma: Proof of Principle, Report of Two Cases

**DOI:** 10.3390/curroncol31090400

**Published:** 2024-09-13

**Authors:** Mathias Oymanns, Michael Daum-Marzian, Chalid Assaf

**Affiliations:** 1 Department of Dermatology, Helios Hospital Krefeld, 47805 Krefeld, Germany; 2 Department of Radiation Oncology, Helios Hospital Krefeld, 47805 Krefeld, Germany; michael.daum-marzian@helios-gesundheit.de; 3 Institute for Molecular Medicine, Medical School Hamburg, 20457 Hamburg, Germany

**Keywords:** mogamulizumab, total skin electron beam therapy, cutaneous T-cell lymphoma, Sézary syndrome, combination therapy, CTCL, TSEBT, mycosis fungoides, CCR4

## Abstract

Patients with advanced-stage mycosis fungoides (MF IIB–IVB) and Sézary syndrome (SS) have poor prognoses, with survival ranging from 4.7 to 1.4 years depending on the disease stage. There is a need for therapeutic approaches that lead to long-lasting responses and improved quality of life and survival. Mogamulizumab, a humanized antibody against the CCR4 molecule, and low-dose total skin electron beam therapy (TSEBT) are two known established treatments for MF and SS as a monotherapy. However, little is known about the potential additive effect on the combination of both treatments. We report here for the first time the concurrent use of low-dose hypofractionated TSEBT (2 × 4 Gy) with mogamulizumab. Based on two relapsed/refractory and advanced-stage CTCL patients, we show that this combination may be well tolerated in advanced-stage MF or SS and may potentially lead to an additive treatment effect on response times, particularly in the skin and blood within two weeks. We propose that this combination may be a treatment option for patients with SS. Further research is needed to understand the efficacy and tolerability profile of this therapeutic combination and to determine if there is an additive effect of the combination on the response rates when compared with the monotherapy.

## 1. Introduction

Primary cutaneous lymphomas are a heterogenous group of non-Hodgkin lymphomas that primarily manifest in the skin [1]. The global incidence is approximately one new case per one-hundred-thousand inhabitants per year, which extrapolates to roughly eight-hundred new diagnoses per year in Germany [2,3]. The World Health Organization-European Organization for Research and Treatment of Cancer (WHO-EORTC) consensus classified multiple subtypes, with the most common cutaneous T-cell lymphoma (CTCL) subtypes including mycosis fungoides (MF, 60%) and Sézary syndrome (SS, 2–5%) [4]. While MF mainly manifests through patches and plaques in the early stages, up to 34% of patients progress to the advanced stages, presenting tumors and erythroderma [1]. SS is an aggressive form of cutaneous lymphoma that is characterized by erythroderma, lymphadenopathy, and the detection of Sézary cells in the skin, lymph nodes, and peripheral blood [1,4].

In the advanced disease stages of mycosis fungoides (stages IIB–IVB) and Sézary syndrome (SS), old age and poor response to initial treatment are some of the key factors correlated with poor prognostic outcomes [5]. The overall survival ranges from 4.7 to 1.4 years for stages IIB to IVB [6]. High remission rates and long-lasting responses are rarely achieved in these patients, especially with the monotherapy, and the treatment options are dependent upon the stage of the disease, particularly for advanced-stage MF or SS [5,7,8]. A multidisciplinary approach, employing various combinations of skin-directed therapies, biologic-response modifiers, and systemic chemotherapeutic agents, is commonplace [5,7,8]. Tolerability, toxicity of polypharmacy, and lack of response are common reasons for treatment failure [5,8]. More effective strategies, such as combination therapies, are needed to enhance the therapeutic responses.

Mogamulizumab is a humanized antibody against C–C chemokine receptor type 4, which initiates antibody-dependent cellular cytotoxicity, and is approved for the treatment of adults with MF and SS after at least one prior systemic therapy [9,10]. Mogamulizumab has shown encouraging response rates and prolonged progression-free survival (PFS) in the phase III open-label, randomized, controlled MAVORIC study when compared with vorinostat [9,10,11,12]. In this study, mogamulizumab (1.0 mg/kg intravenously [IV] weekly for the first 28-day cycle, then on days 1 and 15 of subsequent cycles; n = 186) was shown to have superior PFS in patients with previously treated MF and SS compared with vorinostat (400 mg daily; n = 186). The investigator-assessed median PFS was 7.7 months (95% confidence interval [CI]: 5.7–10.3) compared with 3.1 months (95% CI: 2.9–4.1), respectively (*p* < 0.0001) [12]. Subgroup analyses have demonstrated that mogamulizumab shows increased efficacy in MF/SS patients with higher blood tumor burden (B1/B2) across all the evaluated endpoints, including progression-free survival (PFS), overall response rate (ORR), time to next treatment (TTNT), and modified Severity Weighted Assessment Tool (mSWAT) score [13]. The first published French real-world study, OMEGA, reported promising response rates, with an overall response of 58.7% (SS: 69.5%; MF: 46%) [14].

The most common mogamulizumab-related treatment-emergent adverse events (TEAEs) observed in both phase 3 clinical trials and real-world studies include drug rash, lymphopenia, infusion-related reactions, and fatigue [14]. Mogamulizumab-associated rash (MAR) is believed to result from the activation of macrophages and CD8-positive T-cells and depletion of T-regulatory lymphocytes, although its precise mechanism remains unclear [15,16,17,18]. Interestingly, the occurrence of MAR is associated with improved clinical response rates and overall survival [15,16,17,18,19,20].

Although the post hoc analysis of the MAVORIC data demonstrated a median time to response in the blood compartment (as defined by the global consensus recommendations [8,9]) of 1.1 months, skin response tended to take longer (median 3.0 months), especially in patients with MF and blood involvement, for whom 50% of the skin responses occurred after ≥3.9 months of treatment [13].

Local radiotherapy is highly beneficial for patients with localized skin lesions. However, patients with MF/SS often suffer from widespread skin involvement, affecting more than 10% of their body surface area, which typically necessitates total skin electron beam therapy (TSEBT) instead of multiple localized radiotherapy fields [21,22,23]. The use of TSEBT in Sézary syndrome remains controversial as it yields insufficient long-term remission [24]. Nevertheless, TSEBT has a systemic effect, reducing the circulating Sézary cell counts [24]. For selected SS patients [25], combining TSEBT with immunomodulatory therapy, targeted biologic agents, and subsequent stem cell transplantation offers promising treatment options [26,27]. The potential outcomes of TSEBT in combination with the current immunotherapies include the rapid improvement in the skin symptoms and quality of life within four to eight weeks of RT initiation. TSEBT can also reduce the cutaneous tumor burden (as measured by mSWAT) and lymphadenopathy, especially if initiated before or simultaneously with systemic treatments [28]. In addition, radiotherapy may reprogram the tumor microenvironment (TME), e.g., by upregulating the expression of targeted receptors such as CCR4 [29,30].

Traditionally, conventional fractionated RT with doses ranging from 30 to 40 Gy has been the standard of care for patients with cutaneous lymphomas over the past century. However, in recent years, low-dose TSEBT (8–12 Gy) has often been employed to palliate the cutaneous symptoms of MF and SS, achieving reasonable remission rates [31,32,33,34]. More recently, ultra-hypofractionated low-dose TSEBT, administered in two fractions of 4–8 Gy, has emerged as a valuable treatment protocol for advanced-stage CTCL. This approach aims to achieve rapid remission within days while minimizing toxicity, as well as reducing costs and treatment time [35,36,37,38].

With this background, mogamulizumab and low-dose TSEBT in concurrent combination could have the potential to benefit patients from the outset of treatment by addressing the blood and skin compartments in particular [10,11,39]. This report details two case studies of patients with SS for whom multiple treatments have previously failed due to poor tolerability and lack of response. The patients were both treated with mogamulizumab in combination with concurrent ultra-hypofractionated low-dose TSEBT. The reporting of these case studies conforms to the CARE guidelines [40].

## 2. Patients and Methods

### 2.1. Case 1

A 66-year-old female patient was diagnosed externally with SS in January 2016 and initially presented with suberythroderma with an involved body surface area (BSA) of 70%, pruritus, and lymphadenopathy (Figure S1, Supplementary Materials). The patient had no further relevant medical history. The patient received bath psoralen plus ultraviolet light A and methotrexate 15 mg subcutaneously until January 2018. Due to disease progression, the patient was subsequently treated at our hospital and underwent extracorporeal photopheresis (ECP) every two weeks for 6 months, followed by 4-weekly administration, from January 2018 to June 2020. This initially achieved disease improvement with regression of suberythroderma and pruritus. However, the patient experienced disease progression in June 2020, with progression to erythroderma, pruritus, and increased blood burden.

#### 2.1.1. Clinical Findings and Diagnostic Assessment

In July 2020, due to the patient’s disease progression to erythroderma after prior disease stabilization, blood testing and characterization on fluorescence-activated cell sorting (FACS) were utilized, which revealed a total lymphocyte count of 1035 cells/μL (90% of these lymphocytes were cluster of differentiation [CD]4+/CD7−932 cells/μL) and a CD4/CD8 ratio of 16.4.

#### 2.1.2. Therapeutic Intervention

**Figure 1:**
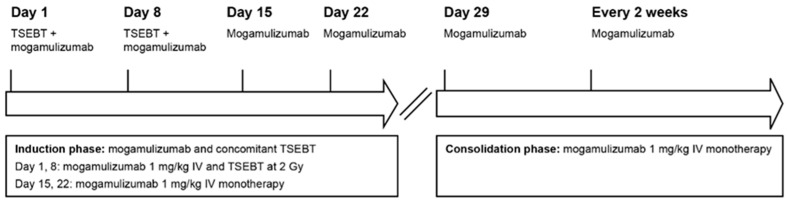
Case 1: Concurrent mogamulizumab and TSEBT regimen. IV, intravenous; TSEBT, total skin electron beam therapy.

Due to disease progression, therapy with mogamulizumab (1 mg/kg IV weekly on days 1, 8, 15, 22, and 29 and then every 2 weeks) was planned in combination with two concurrent administrations of TSEBT at 2 Gy weekly in July 2020 (Figure 1).

#### 2.1.3. Follow-Up and Outcomes

**Figure 2:**
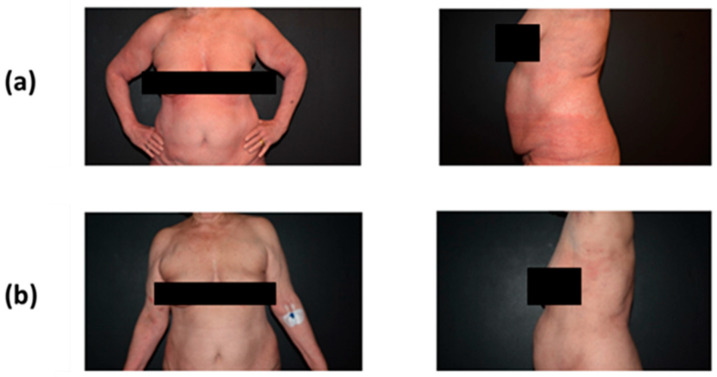
Patient with Sézary syndrome—case 1—(**a**) before and (**b**) after 2 administrations of mogamulizumab and concurrent TSEBT at 2 Gy. TSEBT, total skin electron beam therapy.

Treatment with mogamulizumab was well tolerated with no reported adverse events. After 2 weeks of treatment with mogamulizumab (two doses) and TSEBT, near CR of skin involvement and rapid remission of pruritus were confirmed clinically (Figure 2). In August 2020, a new FACS analysis was conducted after 4 weeks of treatment; this revealed a total lymphocyte count of 488 cells/μL with a CD4/CD8 ratio of 1.5 and a proportion of 4.9% CD4+/CD7– cells, confirming CR in the blood. The patient continues to receive mogamulizumab monotherapy typically every two weeks (as of June 2024, 17 cycles) and has maintained near CR in the skin compartment and CR in the blood (Figure S1, summarized follow-up).

### 2.2. Case 2

This case describes a heavily pretreated 83-year-old male patient who was diagnosed in 2015 with a working diagnosis of SS following initial symptoms of suberythroderma (BSA 80%), pruritus, and lymphadenopathy (Figure S2, Supplementary Materials). FACS analysis revealed a CD4/CD8 ratio of 969, supporting the diagnosis of SS, with a subset of 473/μL CD4+/CD7− lymphocytes and a subset of 230/μL CD4+/CD26− lymphocytes, also indicative of SS (B2). Prior treatments before presentation to our hospital included bexarotene (January 2015–April 2017, dose unknown) as monotherapy and in combination with interferon alpha (June 2015–July 2016, dose unknown) followed by ECP for 3 months (November 2018–February 2019). Subsequent therapies alone were ultimately withdrawn; the patient had a lack of clinical response to chlorambucil (0.4 mg/kg every 2 weeks initially for 4 weeks and 2 mg/day for 2 months, February–April 2019) and developed leukopenia while receiving alemtuzumab (30 mg every 3 weeks, reduced to 10 mg every 3 weeks due to leukopenia, April–July 2019). Brentuximab vedotin (1.8 mg/kg every 3 weeks) was administered between July 2019 and October 2021; the patient achieved disease control for 27 months before experiencing progression of his condition in October 2021.

#### 2.2.1. Clinical Findings and Diagnostic Assessment

The patient relapsed with erythroderma (BSA 90%), scaling, severe pruritus, and extensive palmoplantar hyperkeratosis in October 2021. FACS analyses before the commencement of subsequent treatment showed normal leukocytes with 1200 Sézary cells/mL and a CD4/CD8 ratio of 11.

#### 2.2.2. Therapeutic Intervention

**Figure 3:**
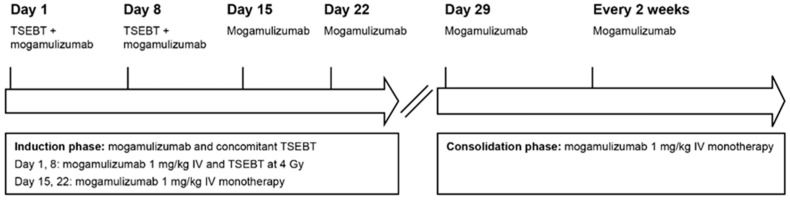
Case 2: Concurrent mogamulizumab and TSEBT regimen. IV, intravenous; TSEBT, total skin electron beam therapy.

Due to disease progression, mogamulizumab was initiated in October 2021 (1 mg/kg IV weekly on days 1, 8, 15, 22, and 29 and then every 2 weeks), administered concurrently with two-dose fractions of TSEBT at 4 Gy weekly (Figure 3).

#### 2.2.3. Follow-Up and Outcomes

**Figure 4:**
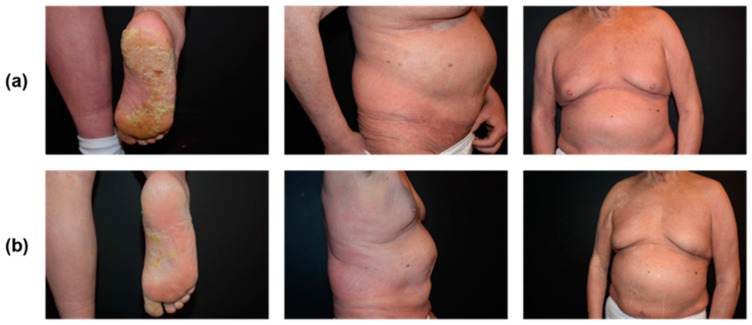
Patient 2 with Sézary syndrome (**a**) before and (**b**) after 11 administrations of mogamulizumab and 2 concurrent administrations of TSEBT at 4 Gy weekly.

Early remission and symptom control were confirmed clinically in the skin within 2 weeks. In addition, blood tumor burden dropped to a CD4/CD8 ratio of 0.49 within this time frame, confirming CR in the blood. Complete clinical remission was maintained for up to 11 administrations of mogamulizumab monotherapy, which was well tolerated (Figure 4). The last administration of mogamulizumab was on 1 March 2022. The patient has been followed up since then and a relapse of the disease with primary skin involvement is currently being treated with a rechallenge of brentuximab vedotin 1.8 mg/kg every 3 weeks. The patient is in partial remission with a good quality of life (Figure S2, summarized follow-up).

## 3. Discussion

In these two patients, the combination of mogamulizumab and concurrent hypofractionated low-dose TSEBT with 2 × 4 Gy for the treatment of SS was demonstrated for the first time. The induction of remission in the skin and good symptom control were achieved within a matter of weeks, with a corresponding CR observed in the blood within 4 weeks. This combination also appeared to be well tolerated during the 2 weeks of treatment with both mogamulizumab and TSEBT.

The MAVORIC trial demonstrated that the median time to response in the blood with mogamulizumab is 1.1 months [15]. This rapid response in the blood was reflected in the above cases, with patient responses within 4 weeks for Case 1 and 2 weeks for Case 2. However, the results from MAVORIC show that the response in the skin following treatment with mogamulizumab typically takes longer than in the blood, at a median of 3.0 months [15]. We propose that the combined effect of mogamulizumab with concurrent TSEBT may lead to a reduction in the time to response in the skin compared with the mogamulizumab monotherapy. Indeed, this potential additive effect may be attributed to the mechanisms of action of these therapies; TSEBT is particularly effective in the skin [41,42], and mogamulizumab works effectively and rapidly against malignant tumor cells, particularly in the blood [15]. Furthermore, a possible alteration in the microenvironment due to radiation and its effect on circulating cells may explain the observed strong response, even though the published data typically show a poor response to TSEBT in patients with T4-SS or advanced-stage MF alone [28,29,40,43]. Whether TSEBT has an influence on the induction of MAR, which is associated with a better clinical response and overall survival, remains elusive [15,16,17,18,19,20].

Therefore, further research on the long-term effects of mogamulizumab with concurrent TSEBT is needed. The data are limited on the combination of mogamulizumab with TSEBT and are currently confined to real-world studies, one of which reported improved outcomes with the mogamulizumab combination treatment (with regimens that included TSEBT) compared with the mogamulizumab monotherapy in 65 patients with MF and SS [42,44]. Therefore, clinical trials are needed to confirm whether this combination leads to a reduced time to response in the skin or any long-term beneficial effects compared with the mogamulizumab monotherapy or TSEBT monotherapy. Indeed, further studies could also elucidate if the combination of mogamulizumab and TSEBT has additive effects or if the therapies work synergistically, as demonstrated for brentuximab vedotin, in which the active component, monomethyl auristatin E, works as a radiosensitizer, enhancing the antitumor effect of the subsequent radiation [45,46]. The translation of this antitumor effect to clinical efficacy was shown in a study of 14 patients with MF or SS who achieved complete or partial remission following the administration of brentuximab vedotin and ultra-hypofractionated low-dose TSEBT (4 Gy) [47]. Furthermore, clinical trials comparing the combination versus the monotherapies of TSEBT and mogamulizumab would enable better understanding the safety profiles of these regimens.

Of note, the clinical responses observed in the two patients described in this manuscript support a recent report by Fong et al. demonstrating the benefit of the combination of mogamulizumab and low-dose TSEBT at 12 Gy [44]. The report describes two patients with SS who received low-dose TSEBT within 2 days after mogamulizumab administration, both of whom exhibited excellent tolerability and good clinical responses. In that report, the two patients showed a global response within 4 and 9 weeks, respectively [44]. Any potential difference in effect between our approach of concurrent treatment by administering mogamulizumab and TSEBT on the same day versus sequential administration on different days as described by Fong et al. cannot be concluded from this limited experience and should be analyzed in further clinical trials. Table 1 shows the comparison of the study details between the Fong et al. study and ours.

Currently, two phase II single-arm clinical trials, (1) a multicenter study led by the European Organisation for Research and Treatment of Cancer Cutaneous Lymphoma Task Force (EORTC-CLTG; NCT04128072 [48]) and (2) a single-center study (Stanford, NCT04256018 [49]), are investigating the combination of mogamulizumab and TSEBT in MF/SS (Table 2). The aim of these trials is to evaluate whether the combination of mogamulizumab and TSEBT has a manageable toxicity profile while controlling the disease burden [48]. The regimens for the EORTC-CTCL study are the standard dosage of mogamulizumab (1 mg/kg IV weekly on days 1, 8, 15, 22, and 29 and then every 2 weeks) for two cycles but with sequential rather than concurrent TSEBT (12 Gy) in eight fractions (four fractions per week), administered after completing mogamulizumab [48]. Conversely, the design of the single-center study from Stanford includes an initial treatment with low-dose TSEBT (12 Gy) over 2–3 weeks followed by the mogamulizumab treatment in the standard dose [49].

Additional studies are required to determine if the dosage of low-dose TSEBT of 12 Gy is necessary or if the hypofractionated form of 8 Gy in two fractions achieves comparable results in disease control and symptom palliation but with greater convenience.

## 4. Conclusions

In conclusion, concurrent therapy with mogamulizumab and hypofractionated low-dose TSEBT was associated with excellent tolerability and promising clinical responses in two patients with Sézary syndrome. This concurrent combination regimen may be a possible treatment option for patients with SS, including those who are heavily pretreated. However, these limited observations should be investigated in further research, including clinical trials, to confirm the efficacy and safety.

## Figures and Tables

**Table 1:** Comparison between the Fong et al. study and ours.

Author	Combination	Evidence	Outcome
Fong S. et al., 2021 [44]	Mogamulizumab + low-dose TSEBT (2 × 12 Gy) (sequential)	Two SS patients (T4N2bM0B2/T4NxM0B2)	Both patients achieved a complete global response (CR) after 3 and 4 cycles (12 and 16 weeks, respectively) following the failure of 4 or 5 prior systemic therapies. The CR is ongoing after 43 and 72 weeks of follow-up
Oymanns M. et al., 2024 [this work]	Mogamulizumab + ultra-hypofractionated low-dose TSEBT (2 × 4 Gy) (concomittant)	Two SS Patients	Both patients achieved a complete response (CR) in the blood and a near CR in the skin during the first cycle (after 2 weeks). The near CR is ongoing after 70 weeks in one patient and was 22 weeks in the other.

**Table 2:** Ongoing trials on the combination therapy of mogamulizumab and TSEBT.

Clinical Trials Number	Combination	Disease Type	Phase	Enrollment (Estimated)	Status	Primary Endpoint
NCT04256018	Mogamulizumab + LD-TSEBT	MF/SS (MF Ib-IV, SS)	Phase 2	30	Recruiting	ORR
NCT04128072 (MOGAT)	Mogamulizumab + TSEBT	CTCL (MF IB-IIB)	Phase 2	43	Recruiting	PFS rate at 48 weeks

## Data Availability

No new data were created or analyzed in this study. Data sharing is not applicable to this article.

## Supplementary Materials

The following supporting information can be downloaded at: https://www.mdpi.com/article/10.3390/curroncol31090400/s1, Figure S1: Timeline of case 1. The historical and current timeline of interventions and outcomes; Figure S2: Timeline of Case 2. The historical and current timeline of interventions and outcomes.
