# Supplementary material for: Mogamulizumab and Concomitant Hypofractionated Low-Dose Total Skin Electron Beam Therapy (2 × 4 Gy) in Cutaneous T-Cell Lymphoma: Proof of Principle, Report of Two Cases
Source: Curr Oncol. 2024 Sep 13;31(9):5412–21. doi: 10.3390/curroncol31090400 (PMC11430877; doi:10.3390/curroncol31090400)
Supplement: Supplementary file 1 [file curroncol-31-00400-s001.zip › curroncol-3175535-supplementary.pdf]

Supplementary Materials:

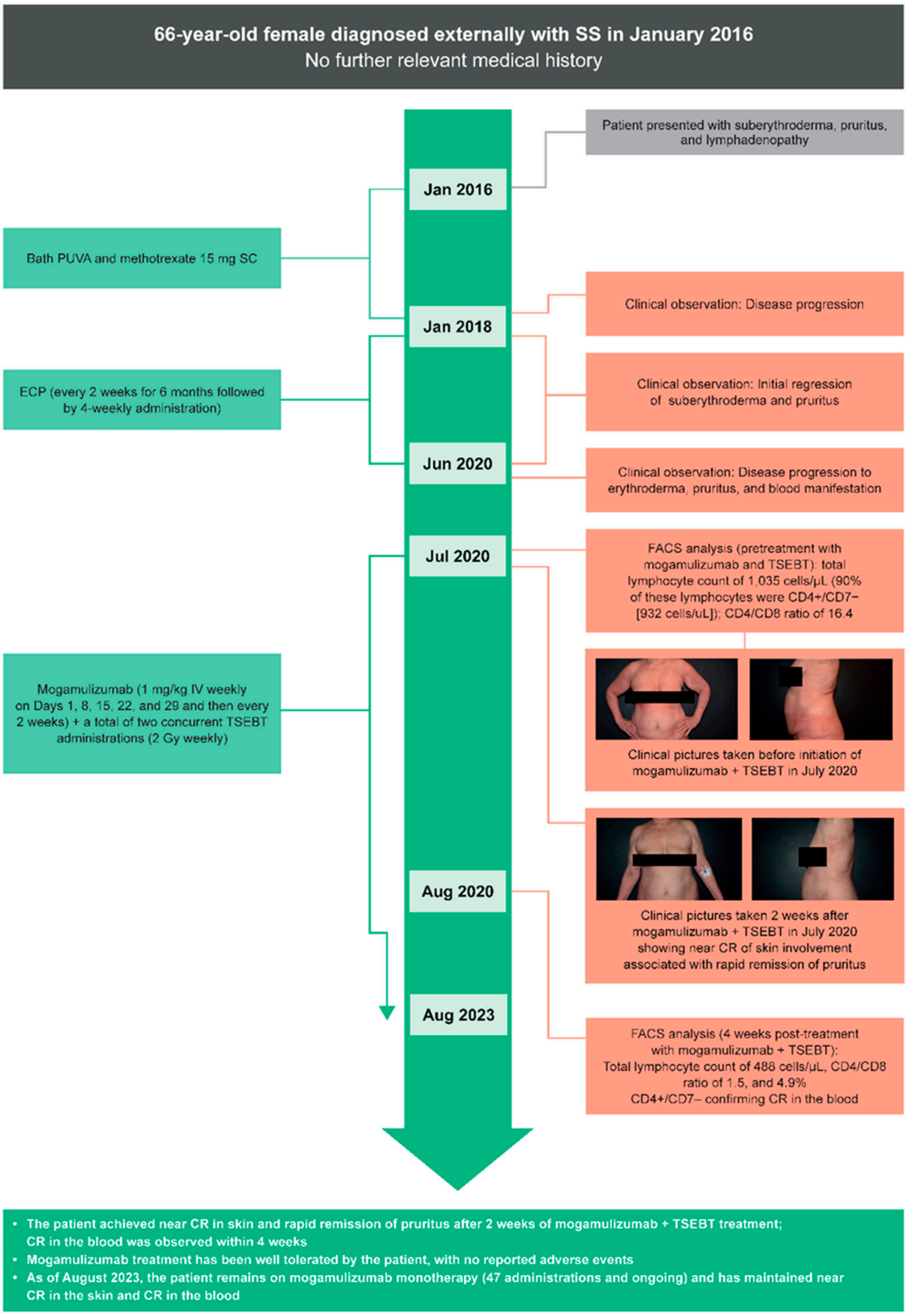

Figure S1. Timeline of case 1. The historical and current timeline of interventions and outcomes.

CD, cluster of differentiation; CR, complete remission; ECP, extracorporeal photopheresis; FACS, flu- orescence-activated cell sorting; IV, intravenous; PUVA, psoralen plus ultraviolet light A; SC, subcutane- ous; SS, Sézary syndrome; TSEBT, total skin electron beam therapy.

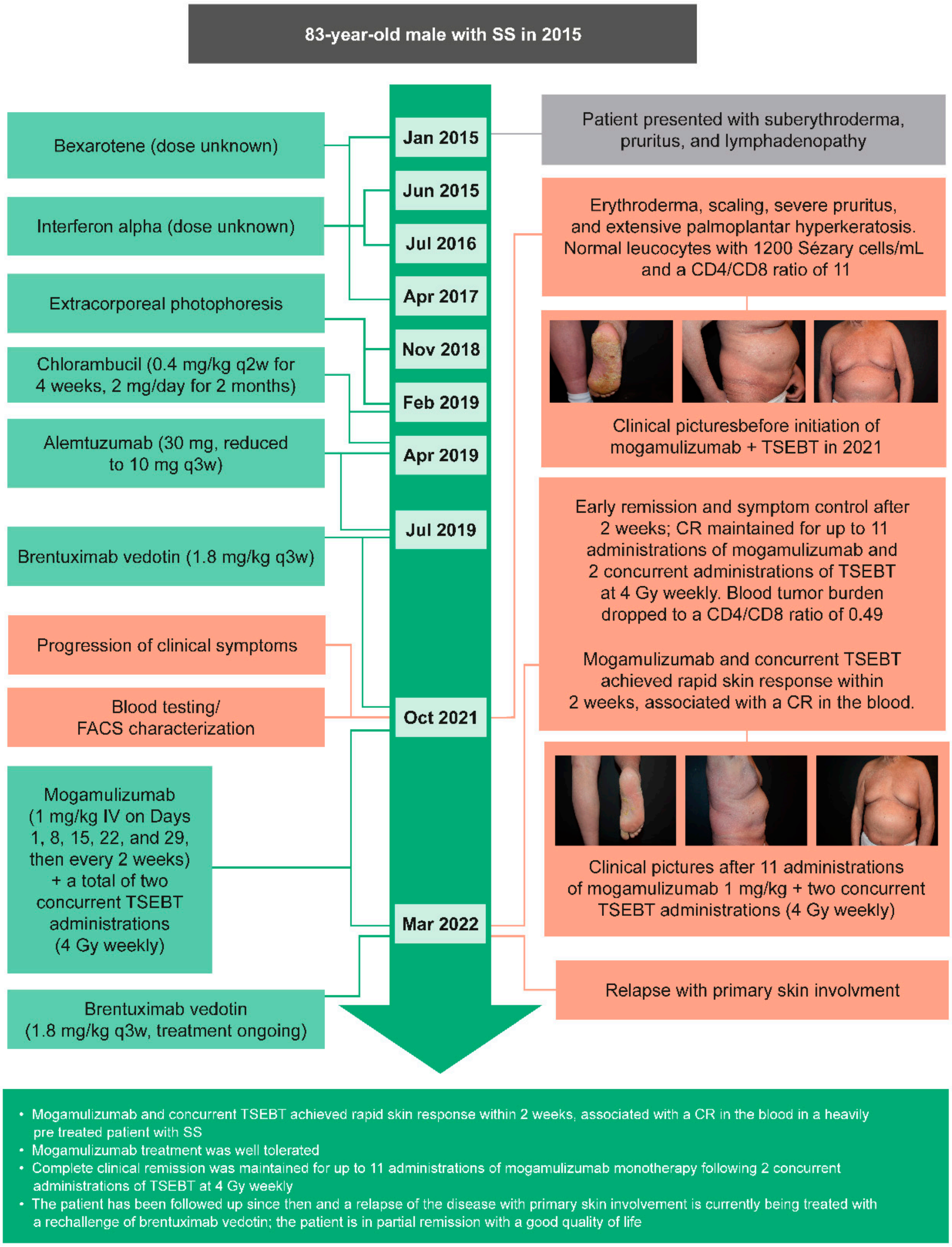

Figure S2. Timeline of Case 2. The historical and current timeline of interventions and outcomes.

CD, cluster of differentiation; CR, complete remission; FACS, fluorescence-activated cell sorting; IV, intravenous; qXw, every X weeks; SS, Sézary syndrome; TSEBT, total skin electron beam therapy.
